# Career mobility of maternal care providers in Mali: a mixed method study on midwives and obstetric nurses

**DOI:** 10.1186/s12960-019-0434-9

**Published:** 2019-12-05

**Authors:** Cheick Sidya Sidibé, Ousmane Touré, Laurence Codjia, Assa Sidibé Keïta, Jacqueline E. W. Broerse, Marjolein Dieleman

**Affiliations:** 1 Institut National de Formation en Sciences de la Santé, Bamako, Mali; 2 Vrije Universiteit Amsterdam, Amsterdam, Netherlands; 3 Department of Health Workforce, Head Quarters World Health Organisation (WHO), Geneva, Switzerland; 4 Centre de Recherche, d’Etudes et de Documentation pour la Survie de l’enfant (CREDOS), Bamako, Mali

**Keywords:** Midwives, Obstetric nurses, Mali, Career mobility, Rural areas, Sages-femmes, Infirmières obstétriciennes, Mali, Mobilité de carrière, Zone rurale

## Abstract

**Background:**

An important strategy to reduce maternal and child mortality in Mali is to increase the number of deliveries assisted by qualified personnel in primary care facilities, especially in rural areas. However, placements and retention of healthcare professionals in rural areas are a major problem, not only in Mali but worldwide, and are a challenge to the health sector. The purpose of this study was to map the mobility of midwives and obstetric nurses during their work lives, in order to better understand their career paths and the role that working in rural areas plays. This article contributes to the understanding of career mobility as a determinant of the retention of rural health professionals.

**Methods:**

A mixed method study was conducted on 2005, 2010, and 2015 cohorts of midwives and obstetric nurses. The cohorts have been defined by their year of graduation.

Quantitative data were collected from 268 midwives and obstetric nurses through questionnaires. Qualitative data had been gathered through semi-structured interviews from 25 midwives and stakeholders. A content analysis was conducted for the qualitative data.

**Results:**

Unemployment rate was high among the respondents: 39.4% for midwives and 59.4% for obstetric nurses. Most of these unemployed nurses and midwives are working, but unpaid. About 80% of the employed midwives were working in urban facilities compared to 64.52% for obstetric nurses. Midwives were employed in community health centers (CSCom) (43%), referral health centers (CSRef) (20%), and private clinics and non-governmental organizations (NGO) (15%). The majority of midwives and obstetric nurses were working in the public sector (75.35%) and as civil servants (65.5%). The employment status of midwives and obstetric nurses evolved from private to public sector, from rural to urban areas, and from volunteer/unpaid to civil servants through recruitment competitions. Qualitative data supported the finding that midwives and obstetric nurses prefer to work as civil servant and preferably in urban areas and CSRef.

**Conclusion:**

The current mobility pattern of midwives and obstetric nurses that brings them from rural to urban areas and towards a civil servant status in CSRef shows that it is not likely to increase their numbers in the short term in places where qualified midwives are most needed.

## Résumé

**Contexte:**

Une stratégie importante de réduction de la mortalité maternelle et infantile au Mali est l’augmentation du taux d’accouchement par du personnel qualifié dans les structures de premier niveau notamment en milieu rural. Toutefois, le recrutement et le maintien en poste des professionnels de la santé dans les zones rurales constituent un problème majeur au Mali et dans le monde et constituent un défi pour le secteur de la santé. Le but de cette étude était de cartographier la mobilité des sages-femmes et des infirmières obstétriciennes au cours de leur vie professionnelle, afin de mieux comprendre leur cheminement de carrière et le rôle que joue le travail dans les zones rurales.

**Méthodes:**

Une étude de méthode mixte a été menée. Les données quantitatives ont été collectées auprès de 268 sages-femmes et infirmières obstétriciennes des cohortes de 2005, 2010 et 2015. Les données qualitatives ont été recueillies lors d'entretiens semi-structurés auprès de 25 sages-femmes et de parties prenantes. Des analyses de statistique descriptives et bi variées ont été réalisées pour les données quantitatives. Une analyse de contenu a été réalisée pour les données qualitatives.

**Résultats:**

Le taux de chômage était élevé parmi les répondants : 39,4% pour les sages-femmes et 59,4% pour les infirmières en obstétrique. Plus de 80% des sages-femmes travaillaient dans des centres urbains contre 64,52% des infirmières obstétriciennes. Les sages-femmes étaient employées dans les CSCom (43%), les CSRéf (20%), les cliniques privées et les ONG (15%). La majorité des sages-femmes et des infirmières obstétriciennes travaillent dans le secteur public (75,35%) et en tant que fonctionnaire (65,5%). Le statut des sages-femmes et des infirmiers obstétriciennes évolue du secteur privé vers le secteur public, des zones rurales aux zones urbaines et de volontaire à fonctionnaire. Les données qualitatives corroborent la conclusion selon laquelle les sages-femmes et les infirmières obstétriciennes préfèrent travailler en tant que fonctionnaire, de préférence dans les zones urbaines dans un CSRéf.

**Conclusion:**

les schémas de mobilité actuels des sages-femmes et des infirmières obstétriciennes qui les amènent des zones rurales aux zones urbaines et le statut de fonctionnaire dans des CSRéf montrent qu'il est peu probable que leur nombre augmente à court terme dans les endroits où les sages-femmes qualifiées sont les plus nécessaires.

## Background

In Mali, maternal mortality is estimated at 368 deaths per 100 000 live births [1]. This ratio is significantly higher than the 70 per 100,000 live births targeted by the Sustainable Development Goals [2]. Because of the lack of human resources for health (HRH) and their unequal distribution among others, the health system in Mali has difficulties to respond to the needs of the population, especially in area of maternal care [3–5]. One of the key strategies to reduce maternal and child mortality is to increase the number of deliveries assisted by qualified personnel in primary care facilities, especially in rural areas [6, 7]. Such facilities are comprised of Community Health Centers (CSCom), referral health centers (CSRef), and private clinics. CSCom offer the minimum package of curative, preventive, and promotional care activities. CSRef offer first referral care and provide technical support to CSCom. Two types of front-line health professionals, namely obstetric nurses and midwives, along with support staff (community midwives) (Cf Table 1), usually care for pregnant women and newborns in primary health care facilities. Obstetric nurses normally perform duties as delegated and supervised by a health care provider, usually a physician or a midwife. They provide perinatal care. Their roles may include conducting normal deliveries where midwives are not available. Midwives provide perinatal care, manage normal deliveries, and detect and refer complications. The number of midwives and obstetric nurses trained in Mali has substantially increased in the past years (from 446 in 2010 to 1538 in 2017) with the establishment of private training schools [5].

**Table 1:** Overview of the training of midwives and obstetric nurses

Health workers	Training duration	Entry level on training	Annual number of graduates	Estimate number of training school	Training sector	Diploma at the end of training
Midwives	3 years	Baccalaureate	About 300	30	Public Private	Yes
Obstetrician nurses	3 years	DEF	About 1200	80	Private	Yes

Midwives and obstetric nurses are employed in the public sector or in the private sector or are self-employed. The government is by far their largest employer (87.6%) [5]. Health workers are recruited through central level government or directly by private facilities. Another way to recruit health workers for public hospitals, CSRef, and CSCom is to use cost recovery funds (so-called ASACO funds for CSCom). Government alternatively also recruits health workers for CSRef and CSCom using funds generated from the so-called HIPC-funds, which is a debt cancellation initiative for the highly indebted poor countries [5]. The intended effects of these different recruitment strategies are to increase the availability of human resources. But the annual level of recruitment remains insufficient to meet the needs, and the distribution of midwives and obstetric nurses across the health system is greatly imbalanced. On an average, 75.6% of midwives in any region are located in urban areas [5].

Conditions that contribute to this problem include negative work environments, weak human resources’ regulatory and management systems, and limited career advancement opportunities [8–11]. In line with WHO’s recommendations to improve the attraction and retention of health workers in “rural and remote areas” [12], the Malian Ministry of Health (MoH) developed a policy and strategic plan for HRH in 2009 [13, 14]. This included attracting and retaining midwives in primary rural facilities. So far, no improvements in the retention of midwives and obstetric nurses in rural areas have been reported [15]. It is possible that obstetric nurses are more likely to be willing and interested to start their career in rural areas and remain working in these areas over time. But available descriptions of midwives’ and obstetric nurses’ career pathways have been scant. This study examines the characteristics and career pathways of midwives and obstetric nurses in Mali. It seeks to gain insight into their career mobility during their work life in order to help develop effective recruitment and retention strategies for these maternal care providers.

Career mobility refers to job changes for individuals in the course of their work lives [16, 17]. These changes take place either between organizations (external mobility) or within organizations (internal mobility) [17], with either a change in responsibilities (vertical mobility) or keeping the same responsibilities (horizontal mobility).

The concept of career mobility has been developed and used primarily in high-income countries [18–20]. While we applied this concept to the health sector in Mali to study the vertical and horizontal mobility of midwives and obstetric nurses, we needed to develop its parameters further to befit the context. External mobility was defined as moving between the public and private sectors. Internal mobility was defined as moving between public posts from rural to urban areas, or the other way around, while having the same employer (e.g., the government). For instance, unemployed midwives and obstetric nurses often decide to work as volunteers in health facilities and provide maternal care but do not receive a salary. Their movement to a paid position was considered internal mobility when it occurred with the same employer (e.g., public sector) and external when with a different employer. Vertical mobility was defined as changing job with increased or decreased responsibilities.

Healthcare workers that experience limited upward movement in their career trajectory may choose to leave their post and may leave the healthcare jobs. This may be of concern in the context of retention [21–25]. Understanding occupation of posts at different stages during a career and the length of time spent at each post provides information on the stability of the HRH workforce. This insight can assist policymakers in setting policies in line with the work-life stages of midwives and obstetric nurses, thus enabling them to develop attraction and retention policies for rural areas, while taking into account the preferences of the two cadres at different stages of their work life. In this study, we aimed to map the mobility of midwives and obstetric nurses during their work lives, in order to better understand their career paths and the role working in rural areas plays in this. This article contributes to the understanding of career mobility as a determinant of the retention of rural health professionals. The study results aim to inform health workforce policies to improve maternal health care services at the primary level, and especially in rural areas in Mali.

## Methods

A mixed method study was conducted. Data collection took place from October to December 2017 in order to answer the following two research questions:

i. What are the midwives’ and obstetric nurses’ employment sectors, workplaces, and duration at a post?
ii. What types of jobs and functions are occupied by midwives and obstetric nurses and with what responsibilities over time?

### Study population and sampling

The study covered the 2005, 2010, and 2015 cohorts of midwives and obstetric nurses from public and private health training schools in order to include midwives and obstetric nurses at different stages of their careers. The cohorts have been defined by their year of graduation. Employers and managers were included from different levels in the health system in three regions in Mali as key informants.

For the quantitative component, graduates from the list of graduates provided by the national institute for training in health sciences (INFSS) were contacted and invited to participate. Those who were reachable and agreed to participate have been included. All respondents were recruited for the study through the INFSS’ schooling department that has the mandate of the government to manage the data of all graduated students in the country. It was possible to contact 31/150 graduates of the 2005 cohort, 130/409 for the 2010 cohort, and 107/548 for the 2015 cohort. All respondents were contacted beforehand by the school records managers to request their participation. All contacted graduates agreed to participate.

For the qualitative component, we selected respondents on the basis of their actual employment status (having a job or not) and their current location (rural or urban). Potential respondents were approached beforehand by the record managers from the school. Rural areas, in this study, are defined as areas located outside main cities and Bamako.

Key informants were people in charge of recruiting, deploying, or managing the employment of midwives and obstetric nurses. Sampling for key informants was done purposively on the basis of the effective involvement in the management.

### Data collection methods and process

For the quantitative component, data collection was done through a survey by telephone after agreeing with the respondents on the appropriate time for a call. Questions were asked about current employment (status, setting, location), work history, current duties, and responsibilities (patient care, coaching, administration, and office tasks and roles).

For the qualitative component, respondents were visited at their place of residence or at their workplace for a semi-structured interview using an interview guide developed and tested with key informants before data collection. Themes explored were career choices, job search strategies, and mobility. For the managers, the same principle was applied. The interviews were semi-structured, and questions were asked about vacancies, strategies to fill posts in rural areas, and health workers’ mobility. Interviews lasted on average 45 min and were conducted by CS who also did the transcriptions and translation.

### Data processing and analysis

Quantitative data were analyzed with IBM SPSS Statistics version 24 using descriptive statistics to identify characteristics of participants. The chi-square test and the Fisher exact test were used for bivariate analysis. The *P* value below 0.05 was considered statistically significant.

Semi-structured interviews were tape recorded in French. They were transcribed and then analyzed with Atlas.ti qualitative software version 8.1.0. A thematic analysis was done. We developed a coding framework based on the research questions. The transcripts were coded and analyzed, comparing and contrasting answers between different groups of respondents. The analysis was undertaken by CSS, and preliminary findings were discussed with MD.

## Results

We present data mainly on midwives. Data on obstetric nurses are reported when there are significant differences.

### Demographic characteristics

The quantitative study included 268 workers of which 136 were obstetric nurses and 132 were midwives. The average age of midwives was 32.4 ± 5.5 years (see Table 2). The number of respondents of the qualitative component was 25 with age ranging from 25 to 45 years (see Table 3). The average duration of having worked as manager was 5 years, and number of years spent within the health system was 8.5 years.

**Table 2:** Sociodemographic characteristics and employment status of respondents in the quantitative component

	Midwives		Obstetric nurses	
	*n*	%	*n*	%
Age				
Mean	32.41		31.23	
Std. deviation	5.5		6.4	
Marital status				
Married	106	80.3	111	81.6
Never married	17	12.9	21	15.4
Divorcee	6	4.5	2	1.5
Widows	3	2.3	2	1.5
Employment situation				
Employed	80	60.6	62	45.6
Unemployed/volunteer	52	39.4	74	54.4
Employed personnel				
Employment sectors				
Public	59	73.75	48	77.42
Private	21	26.25	14	22.58
Structures of employment				
CSCom	34	42.50	28	45.16
CSref	16	20.00	9	14.52
Clinic	12	15.00	8	12.90
Hospital	5	6.25	11	17.74
NGO	3	3.75	4	6.45
Others^+^	10	12.50	2	3.23
Place of work				
Bamako capital town	48	60.00	21	33.87
Region/urban areas	16	20.00	19	30.65
Rural areas	16	20.00	22	35.48
Average duration at a post (years)				
Position 1	1.94 ± 1.76		1.79 ± 2	
Position 2	1.8 ± 1.6		2.3 ± 2.3	
Tasks and responsibility				
Care	69	86.25	59	95.16
Training	9	11.25	0	0
Administration	2	2.5	2	3.23
Employment status/contract				
Civil servant	33	41.25	23	37.10
Community officer	21	26.25	16	25.80
Employee/private contract	20	25.00	16	25.80
HIPC Fund	5	6.25	3	4.84
Asaco Fund	1	1.25	4	6.46
Have a signed contract (private sector)				
Yes	7	33.33	11	55.00
Unemployed/volunteer personnel				
Number of positions employed as volunteer				
1–2	20	39.20	39	57.35
≥3	31	60.80	29	42.65
Average duration at a post (year)				
Position 1	1.44 ± 1.14		1.36 ± 1.25	
Position 2	1.5 ± 1.6		1.18 ± 1.1	
Assignment/volunteer location				
Bamako capital town	33	68.75	41	63.08
Region/urban areas	4	8.34	8	12.30
Rural areas	11	22.91	16	24.62
Facilities				
CSCom	22	45.83	25	38.4
CSRef	18	37.50	21	32.30
Hospital	7	14.58	8	12.30
Private clinic	0	0	6	9.23
Others^a^	1	2.09	5	7.70

^a^School, faith-based structure

**Table 3:** Characteristics of respondents in the qualitative component

	Obstetric nurses	Midwives	Key informants
Cohort			
2005	2	2	
2010	3	6	
2015	3	3	
Other			6
Age range (years)	25–35	25–35	35–45
Marital status			
Married	7	11	6
Single	1		
Location of employment			
Urban areas	4	6	3
Rural areas	4	5	3
Status of employment			
Civil servant	8	11	4
NGO/Private fund			1
Community health association			1
Employment sector			
Public	8	11	6
Private			

### Current employment status

The employment rate was 100% (*n* = 20) for the 2005 cohort at the time of the data collection, 79.7% (*n* = 47) of the graduates of the 2010 cohort had a paid-job 8 years after graduation, and 24.5% (*n* = 13) of the 2015 cohort had a paid-job 2 years after graduation. The jobs (paid and unpaid) held by midwives were typically, first as a care provider (86.25%, *n* = 69), followed by a trainer (11.25%, *n* = 9). None of the obstetric nurses were employed as trainers. Most of the midwives (73.75%, *n* = 59) and obstetric nurses (77.42%, *n* = 48) were employed in the public sector (Cf Table 2).

Most midwives, about 80% (*n* = 64), worked in urban facilities compared to 64.52% for obstetric nurses (*n* = 40) (*p* = 0.008). Midwives were employed in CSCom (43%, *n* = 34), CSRef (20%, *n* = 16), and private clinics and NGO (15%, *n* = 12). The same employment structures are found for the obstetric nurses (see Table 2).

Among the respondents, 39.4% (*n* = 52) of midwives were unemployed or volunteers. Of these, most (68.76%, *n* = 33) worked as volunteers in Bamako, and 22.91% (*n* = 11) worked as volunteers in rural areas. Of those who are unemployed or volunteer, 95.35% (*n* = 41) were actively searching for a job.

According to the semi-structured interviews, voluntary employment had two main objectives: to maintain and strengthen skills and to keep in touch with the world of employment so as to be informed about opportunities and job offers.

I came to the CSCom to work as a volunteer. There was HIPC recruitment, the doctor informed me and demanded me to apply. He saw that I was working well. He demanded them to assign me in CSRef when I was selected. (Midwife 2010)

### Strategies to find employment

According to the interviews, a key strategy used by midwives to find jobs in the public sector was competition through a written test. In the private sector, they would actively approach and ask managers at facilities. Students of the same cohort and institution organized themselves into information groups by phone and via social networks to keep in touch and to stay informed. They also searched through the newspaper and Internet advertisements. Research strategies, jobs sought, and employment sectors chosen were influenced by parents and spouses, in addition to their own preferences.

My husband supported me in my job searches. But he does not want me to work in NGOs. I do not know what is in the NGOs or why he does not want me to work there. (Midwife 2010)

Reported difficulties in finding a job were related to not knowing where to search, the frequent follow-up of applications required, and the high number of candidates especially for public services.

According to managers at the district level, there are vacancies in rural areas for midwives, but they have little interest for these posts.

### Mobility of maternal care providers

The average number of positions held by midwives (all cohorts combined), from the moment they started to work, was 3 ± 1.4. The average duration in a post was 1.9 ± 1.7 years.

The main reasons for job changes for civil servants were transfer on request to join the family (80%), studies (10%), and insecure living and working areas (10%). For those who were employed on HIPC funds or on Asaco funds, the main reasons to change were not being interested in working for Asaco or under HIPC funds due to the salary level (50%) and job insecurity (38%). Those working in the private sector changed jobs because of not prolonging contracts, being fired or closure of projects (39%), not wanting to continue in the private sector (25%), or joining their family (13%). In order to understand the mobility of midwives, an example of a typical career path of a midwife in Mali is described in Table 4.

**Table 4:** An example of a typical career path of a midwife in Mali

Midwife, Promo 2005. Civil servant	
I have been a midwife since 2005. I was employed on HIPC funds and then I became a civil servant. I had my first job in 2007. In the meantime, I had taken a volunteer position at a maternity hospital in 2006 after hitting several doors to have a job in Bamako, but without success. I stayed at this position for about one year. I accepted because it was close to my home.	
In 2007 I had the job on HIPC funds. I was informed by a friend of a post available in rural areas. She did not like it because she did not like working in rural areas. I said yes because I could not wait to get a job. It was in a CSRef in the Koulikoro region. There I stayed for two years. From there I did the public service competition of which I was informed by friends. After admission I was posted to a CSCom in the Mopti region as a civil servant. There I was the manager. I stayed there for two years too. Then I came here to Ségou region.	
My changes were for family reasons and the problem still persists because my husband works elsewhere. When I was admitted to the public service my chief physician did not want me to be transferred. But I had been assigned to Mopti region and my husband was in the Segou region. I asked for a transfer to join him. The transfer came out and my husband had a job elsewhere and my transfer remained in Segou. My problem was not solved. I still wanted to join him. My mother then advised me to stay and to stop the shuttling back and forth which prevented me from working for my country which trained me for so many years. At the moment I have decided to stay, and my husband has also understood.	
Coming here to the CSRef, as I was the most experienced midwife, I have been appointed midwife mistress and also received training which helped me understand a little more about administration.	

#### External and internal mobility

The findings show that in terms of external and internal mobility, there is a move among maternal care staff from volunteerism to employment status and from rural to urban with the same employer (internal mobility pattern) and from private sector to the public sector (external mobility pattern).

##### External mobility pathway

**Fig. 1:**
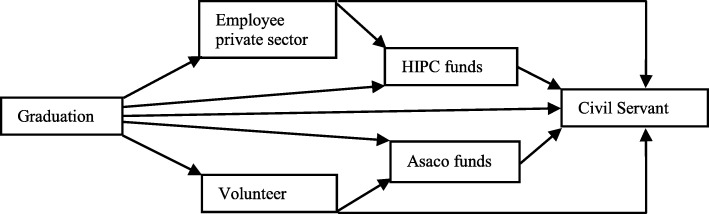
Evolution of midwives’ employment status

At their first job, 60% (*n* = 48) of midwives, all cohorts combined, were volunteers; 18.75% (*n* = 15) were employed in private sector; 11.25% were civil servants (*n* = 9); and 10% were employed on HIPC or Asaco funds (*n* = 8). The status of a civil servant seems to be the final status sought, as 67.5% (*n* = 54) of midwives became civil servants after several years. According to our data, the evolution of the career for midwives seems in general to start as volunteer and to be finally employed as a civil servant. Very few were employed on Asaco funds during their career. Figure 1 reflects what we have found in the study.

With respect to working in the public or private sector, mobility was from private to public. Midwives working in the private sector or for Asaco were more likely to leave their position and job than those of the public sector (*p* = 0.02). No case of transition from public to private sector was found.

##### Internal mobility pathway: movement between rural and urban areas

**Fig. 2:**
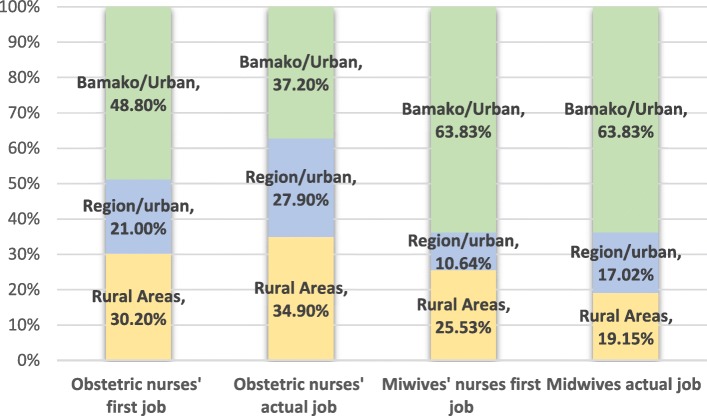
Evolution by area of employment, cohort 2010

Midwives were more likely to move to or stay in urban areas, mostly Bamako (see Fig. 2).

Qualitative data shows that the changes of employment location have two main reasons: family reasons and professional reasons, namely searching for centers with sufficient workload to maintain skills or searching for training opportunities.

There were things I wanted to do as a professional that I could not do there. My colleagues in towns could do more than me. I asked to come in town, so I could have the chance to do some of these things and to keep up my skills. My requests were motivated by the fact that there was not enough work, I wanted to go to town to work more. (Midwives, 2010)

The midwives also asked to leave after about 3 years when they felt they had spent sufficient time in a rural area. Managers confirmed these reasons and added that the most important reason for requests for transfer were related to family. They also asserted that midwives would sometimes express frustration when they are in a community which prefers community midwives who come from these communities. They also may have the feeling of being forgotten when new recruits have jobs in urban areas or are transferred from rural to urban areas. There is also the reason that in rural areas, accessing information and training opportunities is more difficult than in urban areas.

For example, to compete for jobs or better positions, they often are aware at the last minute and therefore too late to be prepared or to apply and they have to give up. (DTC, rural CSCom)

##### Horizontal and vertical mobility: evolution of tasks and responsibilities

The findings show that there is mainly horizontal mobility: Midwives’ tasks and responsibilities varied little during their professional life. Respondents in interviews mentioned that when there are fewer staff members, especially in rural areas, they are responsible for all maternity care; sometimes, they even head facilities in the absence of a doctor, regardless of their qualification.

I stayed in this job for seven years. The first two years were at the maternity ward. After three years I was head of post. There was no doctor, so I was assuming all the responsibilities. (Obstetric nurse, 2005)

Those who are employed in urban and better staffed centers have more specific tasks. Midwives are able to progress to Unit Leader and Midwife in charge. These responsibilities, based on seniority, do not result in a change of tasks, but often coordination and administrative tasks are added.

Since I started working, I have never changed. I do prenatal, postnatal care, deliveries, reproductive health care. Now I am the head of the midwives. When the former was assigned to the Regional Health Department, I was the oldest midwife, and I was appointed. (Midwife, 2005)

## Discussion

It is important to assure quality maternal care for rural populations and provision of maternal health services by qualified personnel, and in particular by midwives, at the primary level and in rural areas [26]. But in 2016, only 23% of midwives were present at rural care services [15]. Strategies to attract and retain midwives in rural and remote areas, and at the primary level, include focusing on training, recruitment, deployment, and retention [12]. Such strategies for training, recruitment, and retention have been developed in Mali [13, 14, 26, 27], but to date they have not been evaluated and results are unknown. Our study seems to indicate that these policies did not lead to an increased interest among midwives to work in rural areas. Moreover, it also shows that the current situation for midwives in Mali does not allow successful fulfillment of the ambition to employ midwives in all rural primary care facilities despite the current high unemployment rate among midwives.

This study intended to explore the mobility of midwives and obstetric nurses in Mali. It shows that the most prevalent type of mobility is horizontal, both internal and external: midwives move from private to public sector and from rural to urban areas.

### External horizontal mobility: movement between private and public sector

Voluntary work in health facilities (private or public) without a salary is for most midwives and obstetric nurses important as it allows them to maintain their skills, to acquire work experience, and to be informed about potential job openings. In order to be recruited, health professionals in Mali generally have to do written tests and the curriculum vitae is not consulted. Therefore, voluntary work does not facilitate getting a paid job, especially in the public sector.

Our study showed that currently midwives as well as obstetric nurses (paid and unpaid) aim to work as civil servants in the public sector. This may be attributable to a number of factors. Firstly, the private sector in Mali is not developed, and nearly all private facilities are located in Bamako. A report from the human resources’ directorate indicates that private facilities do not recruit many midwives [5]. When they do recruit, the salary is less than in the public sector (40 000f CFA versus 110 000f CFA in public sector). Secondly, the public sector provides job security and guarantees access to various benefits like pension schemes and insurances that are missing in the private clinics. For instance, the results show that not all private facilities had signed contracts with employees. In such a condition, the private sector is not likely to attract and to contribute to the availability of midwives and obstetric nurses in primary level care services in rural areas.

### Internal horizontal mobility: movement between areas of employment and type of facilities

According to our survey, midwives (paid and unpaid) are currently looking for health facilities in areas that provide good living and working conditions and, as observed in low-income countries, are moving from rural to urban areas and within government-held structure [21, 28–31]. On the other hand, obstetric nurses tend to be more present in rural areas and to move out of Bamako. Attraction to or retention in rural areas is said to be influenced by personal characteristics, availability of equipment, living conditions, and professional development opportunities [8]. In addition, gender plays an important role in Mali. Most midwives are women and social codes for marriage and family make it obligatory for women to follow the movements of their spouse despite current policies to improve retention. In this context, new retention policies only aimed at midwives are not likely to be effective. A sustainable rural area staffing strategy must be developed within a broader intersectoral collaboration between ministerial departments responsible for labor, health, and social affairs [32]. Moreover, it must take into account existing regulations and social norms.

### Vertical mobility: changes in responsibilities

Vertical mobility that encompasses changing in level of responsibility is not common for midwives according to our findings. They do not usually move to occupations that require higher education. The most common other occupation categories that midwives moved to were administrative and teaching occupations. When they move from private to public or from rural to urban areas, these moves are usually not with promotion. They will be assigned to the same roles and responsibilities and the same type of post. When in rural areas they can be the only person in charge, transferred in urban or more staffed structures, the tasks can become more specific and with less responsibilities but with no changes in salary. For obstetric nurses, transfer to Bamako may result in voluntary demotion as they can lose their informal responsibilities of performing deliveries, or of being in charge. This reduction in responsibilities did not seem to be a problem, and in our study, only few respondents showed a preference for upward career movement and an interest in level of responsibilities.

Overall, the study demonstrated that midwives are unlikely to stay in rural areas and most have the ambition to move from rural to urban areas, despite the retention efforts in place. Therefore, it is important to provide an alternative for improvement of the quality of care for women in rural areas. Currently, if CSCom in rural areas have qualified maternal staff, these are mostly obstetric nurses. The cessation of obstetric nurses’ training by the government and the consequent lack of supervision of training institutions will probably lead to a decline in the quality of the obstetric nurses and will further contribute to a deterioration in the quality of care at the peripheral level. If the availability of midwives in rural areas cannot be ensured in the immediate future, the use of obstetric nurses needs to be continued. However, their quality needs to be guaranteed as well. This is only possible if monitoring of obstetric nurses’ training and supervision at the workplace is offered by the Ministry of Health.

## Study limitation

The number of midwives and obstetric nurses in the 2005 cohort was very small due to the difficulty of reaching these graduates. As we were not able to draw a random sample for the survey, it is not a representative sample of all midwives and obstetric nurses in Mali. The results of the study are therefore an indication of the employment situation and mobility of midwives and obstetric nurses.

The non-inclusion of community midwives (support staff) who may be more desirable to local communities and are more likely to remain in the communities where they originated from may also be a potential limitation to interpretation of the results of this study.

## Conclusions

The deployment of midwives in rural and remote areas is likely to improve maternal, child, and neonatal health. But their current mobility patterns are not likely to increase their numbers in the short term where qualified midwives are most needed. Alternatives to improve the quality of maternal care at primary level in rural areas include support for local training, employment, and supervision of obstetric nurses.

## Data Availability

The datasets used and/or analyzed during the current study are available from the corresponding author.

## Abbreviations

Asaco: Community health association
CSCom: Community health center
CSRef: Referral health center
HIPC: Highly indebted poor countries
HRH: Human resources for health
INFSS: National Institute for Training Health Sciences
MoH: Ministry of Health
NGO: Non-governmental organization
SDG: Sustainable development goals
LMD: License Master Doctorate
